# Effectiveness of pharmaceutical support by pharmacists in urinary care teams

**DOI:** 10.1186/s40780-019-0141-7

**Published:** 2019-06-03

**Authors:** Takumi Umemura, Eri Wakita, Masami Asano, Takahito Mizuno, Koji Kozaki, Yoshiaki Ikeda, Hirokazu Takeda

**Affiliations:** 1 Department of Pharmacy, Tosei General Hospital, 160, Nishi oiwakecho, Seto, Aichi 489-8642 Japan; 2 Urinary Care Team, Tosei General Hospital, 160, Nishi oiwakecho, Seto, Aichi 489-8642 Japan; 3 College of Pharmacy, Kinjo Gakuin University, 2-1723, Omori, Moriyama-ku, Nagoya, Aichi 463-8521 Japan; 4 Department of Urology, Tosei General Hospital, 160, Nishi oiwakecho, Seto, Aichi 489-8642 Japan

**Keywords:** Pharmaceutical support, Urinary care team, Pharmacist

## Abstract

To facilitate timely removal of urinary catheters and promote self-voiding among inpatients, urinary care teams have been established in some Japanese medical institutions. However, direct evidence of the effectiveness of pharmacist intervention in urinary care teams is limited. We evaluated the efficacy of pharmaceutical support by a pharmacist in a urinary care team. Between September 2017 and August 2018, 84 patients met the criteria for initiating continuous intervention. Patients with (20 cases) and without (8 cases) adoption of pharmaceutical support (initiation or discontinuation of treatment for dysuria) were scored for urinary function (including degree of independence of urination and score of lower urinary tract disorder) and for urinary situation. Comparative analysis results showed that pharmacist intervention in the adoption cases resulted in significantly improved scores for urinary function than in non-adoption cases. Similarly, pharmaceutical support resulted in improved overall urinary situation in the patients (85.0% of adoption cases compared to 37.5% of the non-adoption cases). The most common pharmaceutical support was a recommendation to discontinue drugs that induce dysuria (65.0% of the cases). Taken together, our findings suggested that pharmacists are important members of urinary care teams.

## Background

Inappropriate long-term indwelling catheterization is common among patients in acute hospital settings. It is a known leading cause of urinary tract infection, cystolithiasis, urethral injury, fistula formation, and erosion of the bladder neck and urinary sphincter [1, 2]. Urinary incontinence in dependent elderly patients is closely associated with impairment in activity of daily living (ADL) and cognitive function [3]. Furthermore, problems associated with urination are major psychological burdens for inpatients [4]. Therefore, timely removal of urinary catheter and promotion of self-voiding are beneficial for inpatients. In addition, the World Health Organization recommends that prompted voiding be offered for older people as a part of urinary incontinence management [5]. To address these issues, urinary care teams have been established in some Japanese medical institutions. Members of the team include a urological physician, a well-trained nurse, and a physical therapist, but not a pharmacist. There are already reports on the effects of continence care for elderly patients [6, 7]. However, direct evidence regarding the effectiveness of pharmacist intervention in a urinary care team is limited. In this study, we evaluated the efficacy of pharmaceutical support by a pharmacist in a urinary care team.

## Methods

### Study samples

In Tosei General Hospital (633 beds), between September 2017 and August 2018, 84 patients met the criteria for starting continuous intervention by the urinary care team comprised of a urological physician, two well-trained nurse, a physical therapist, and a pharmacist (Table 1). The pharmacist in the urinary care team suggested pharmaceutical support for 28 out of the 84 patients. The criteria for pharmaceutical support (Table 2) included the need for appropriate antibiotic therapy, discontinuation of drugs that induce dysuria, and starting medication for dysuria. This study was approved by the ethics committee of Tosei General Hospital (receipt No. 746).

**Table 1:** Intervention criteria for the urinary care team

When one criterion each is met for 1 and 2, team intervention is needed			
1: Anticipated lower urinary tract disorder			
□ History of dysuria [urinary retention, urinary incontinence, or frequent urination (> 15 times per day)]			
□ History of intrapelvic surgery			
□ Admission for neurological or spinal disease			
□ Fulfillment of the criteria for Evaluating the Degree of Independence (degree of “bedriddenness”) of Disabled Elderly Persons in Performing Activities of Daily Living Rank B2, C1, or C2			
2: Assessment in lower urinary tract disorder after evulsion of urethral catheter			
□ Urinary retention			
□ Dysuria (residual urine volume > 50 mL)			
□ Urinary incontinence			
□ Frequent urination (> 15 times per day)

**Table 2:** Criteria for pharmaceutical support

	Item	Criteria
A.	Appropriate antibiotic therapy	If antibiotics are administered for urinary tract infection, we evaluate and suggest their appropriate use based on antimicrobial sensitivity test of blood or urine culture.
B.	Discontinuation of drug that induces dysuria	If the patients receive drugs that induce dysuria, we suggest continuation or discontinuation of the drugs.
C.	Starting drug therapy for dysuria	If the patients do not receive drugs for dysuria, we suggest starting the drugs (a1 blocker, cholinesterase inhibitor, etc.).

### Variables

The following data were obtained for comparative analysis of change in urination independence in cases with adoption (20 cases) and without adoption (8 cases) of pharmaceutical support. The variables were age, sex, primary disease, total score of urinary function, and urinary situation. The total score of urinary function was the sum of the degree of independence of urination and the score of lower urinary tract disorder (Table 3); low scores indicate improved independence of urination, according to the standards of the Japanese Society of Wound, Ostomy and Continence Management, the Japanese Society of Geriatric Urology, the Japanese Urological Association, and the Japanese Continence Society. Improvement in urinary situation was defined as a decrease in times of intermittent urethral catheterization, withdrawal from intermittent urethral catheterization, and improvement in frequency of urination (≦7 times per day).

**Table 3:** Scoring of urinary function

A. Degree of independence of urination	0	1	2
Movement/transfer	Independence	Partial assistance	High assistance
Toilet activity	Independence	Partial assistance	High assistance
Usage of instrument for urination	None or use by self	Partial assistance	High assistance
Use of diaper or pad	None or use by self	Partial assistance	High assistance
Intermittent urethral catheterization	None or use by self	Partial assistance	with continuous urethral catheter or High assistance
B. Lower urinary tract disorder	0	1	2
Desire to void	Yes	Yes (sometimes)	Almost none
Urinary incontinence	No	Sometimes	Almost
Frequency of urination (per day)	≤7 times	8–14 times	≥15 times
Average voided volume (per time)	≥200 mL	100–199 mL	≤99 mL
Residual urine volume	≤49 mL	50–199 mL	≥200 mL
C. Total score of urinary function = A + B			

### Statistical analysis

Qualitative and stratified continuous variables were compared using the Fisher Exact test or Pearson χ^2^ test. Continuous variables were compared using the Mann-Whitney U test. Predictive values are presented as the odds ratios (ORs) with respective 95% confidence intervals (CI). Two-tailed *p* < 0.05 indicated statistical significance. All the analyses were performed using IBM SPSS Statistics ver 25 (IBM®, New York).

## Results

Table 4 shows the results of the univariate analysis of patient characteristics. The most common primary disease was femoral fracture. The median of the total score of urinary function was 13.0 [interquartile range (IQR): 9.5–14.0] and 11.5 (IQR 10.0–13.3) for the adoption and non-adoption groups, respectively. There were no statistically significant differences in all variables between the two groups.

**Table 4:** Patient characteristics

	Adoption *n* = 20		Non- adoption *n* = 8		*p*-value
Age^a^	85.5	(80.0–90.3)	80.5	(77.8–85.6)	0.328^b^
Sex (male/female)	11/9		2/6		0.221^c^
Primary disease					
Femoral fracture	7		4		–
Cerebrovascular disease	3		1		
Heart failure	1		1		
Orthopedics	1		1		
Pneumothorax	1		0		
Prostate cancer	1		0		
Cellulitis	1		0		
Endometriosis	1		0		
Pulmonary embolism	1		0		
Gastritis	1		0		
Heart stroke	1		0		
Fever of unknown origin	1		0		
Aspiration pneumonia	0		1		
Hospital stay duration (days)					
Overall^a^	39.0	(30.0–47.3)	39.5	(31.0–48.6)	0.500^1^
After intervention of urinary care team^a^	22.5	(19.5–31.0)	22.0	(20.0–29.0)	0.636^b^
Score					
Total score of urinary function^a^	13.0	(9.5–14.0)	11.5	(10.0–13.3)	0.746^b^
Degree of independence of urination^a^	8.0	(5.0–10.0)	7.0	(5.5–8.0)	0.381^b^
Score of lower urinary tract disorder^a^	4.0	(3.0–5.0)	4.0	(4.0–6.3)	0.381^b^
Urinary situation					
Intermittent urethral catheterization	17		6		0.391^d^
Urinary incontinence	1		0		
Use of nursing care diaper	1		2		
Continuous urethral catheter	1		0		

^a^Median (interquartile range; IQR)

^b^Mann-Whiteny U test

^c^Fisher Exact test

^d^Pearson χ^2^ test

Table 5 shows the efficacy of pharmaceutical support for patients under the urinary care team. The total score of urinary function and score of lower urinary tract disorder significantly decreased (*p* = 0.049 and *p* = 0.008, respectively) in the adoption group, compared to those in the non-adoption group. Similarly, the adoption group showed more improved urinary situation (17/20 cases, 85.0%) than the non-adoption group (3/8 cases, 37.5%) (*p* = 0.022). Table 6 shows the breakdown of pharmaceutical support in the adoption group. Discontinuation of drugs that induce dysuria (13 cases, 65.0%) was the most common recommendation for pharmaceutical support. Solifenacin succinate and tramadol + acetaminophen were the most commonly discontinued drugs (3 cases each). There was no case for appropriate antibiotic therapy.

**Table 5:** Efficacy of pharmaceutical support in the urinary care team

	Adoption n = 20		Non-adoption n = 8		*p*-value
Score after intervention					
Total score of urinary function^a^	7.5	(5.8–9.3)	10.5	(7–15.3)	0.049^b^
Degree of independence of urination^a^	5.0	(4.0–7.0)	7.0	(4.8–8.6)	0.281^b^
Score of lower urinary tract disorder^a^	2.0	(1.8–3.0)	4.5	(3.8–5.3)	0.008^b^
Urinary situation					
Improvement	17	(85.0%)	3	(37.5%)	0.022^c^
Normal urination		11		2	
Decreased times of intermittent urethral catheterization		5		1	
Use of nursing care diaper		1		0	

^a^Median (interquartile range; IQR)

^b^Mann-Whiteny U test

^c^Fisher Exact test

**Table 6:** Breakdown of pharmaceutical support in the adoption group

Pharmaceutical support	n = 20
Discontinuation of drugs that induce dysuria	13
Starting drug therapy for dysuria	5
Both discontinuation of drugs that induce dysuria and starting drug therapy for dysuria	1
Observation^a^	1

^a^The pharmacist recommended not to start drug therapy for dysuria against the suggestion of the urological physician

## Discussion

The Japanese population is rapidly aging owing to declined birthrate. In 2018, elderly people of at least 65 years of age consisted 28.1% of the Japanese population [8]. In this population, aging-related reduction in intrinsic capacity has led to low quality of life. Among aging-related diseases, urination disorders, which starts from 40 years of age, have shown increasing severity. Urinary catheters are used as first aid for acute urinary retention, as temporary measures for declined renal function and hydronephrosis due to chronic urinary retention, as management of dysuria due to underactive detrusor, and as support for patients who are difficult to treat due to age or other complications [9]. However, long-term indwelling urinary catheters can cause several complications in the lower urinary tract [1, 2]. Appropriate management of urethral catheter has been found to be essential for preventing urinary complications [1, 2, 10, 11]. Therefore, prolonged use of indwelling urinary catheters is not recommended, and a switch to other urination management plans, such as intermittent urethral catheterization, should be made as soon as possible. Urinary care teams have been established in some Japanese medical institutions to facilitate timely removal of urinary catheters and to promote self-voiding among inpatients. However, because the efficacy of pharmaceutical support by a pharmacist as a member of urinary care teams has not been reported, we conducted this study.

In this study, pharmaceutical support by a pharmacist improved urinary situation in 85.0% of the patients and decreased the score of urinary function without of prolongation of hospitalization, compared to that in the non-adoption group (Table 5). These results indicated that pharmaceutical support by a pharmacist was an important addition to the urinary care team.

Drugs such as anticholinergics are among the causes of lower urinary tract symptoms (LUTS), such as voiding and storage symptoms [3]. Middle-aged and elderly persons often have underlying diseases, such as benign prostatic hypertrophy, aging-related detrusor hyperreflexia, and neurogenic bladder, and LUTS are caused by using drugs for these conditions. Moreover, the elderly frequently has combinations of cardiovascular disease, metabolic disorder, neuropsychiatric disorder, and malignancy, thereby experiencing polypharmacy. Hashimoto et al. reported that LUTS-causing drugs were correlated with polypharmacy, and that adverse drug events associated with LUTS-causing drugs were highly prevalent in elderly patients [12]. Therefore, we considered that evaluation and discontinuation of LUTS-causing drugs, where appropriate, may be beneficial for patients. In this study, we recommended drug discontinuation for 70% (14/20) of the cases in the adoption group (Table 6). Our support significantly decreased the score of lower urinary tract disorder, but did not improve the degree of independence of urination (Table 5). These results suggested that pharmaceutical support through discontinuation of certain drugs (Table 2) contributed to the improvement of urinary function, but not of ADL. Furthermore, there was no case that required appropriate antibiotic therapy in this study.

Clean intermittent catheterization is less likely to cause urinary tract infection than continuous urethral catheterization [13, 14]. Therefore, for patients in acute settings, it is ideal to switch from chronic indwelling catheters to clean intermittent catheters as soon as possible. Additionally, frequent intermittent urethral catheterization has been linked to urinary tract infection [3]. Therefore, reduced frequency of intermittent catheterization may reduce risks of complications in addition to improving the overall urinary situation of the patients. Generally, to reduce the frequency of intermittent catheterization, the urinary care team participated in nursing care, such as by periodically inducing patients to go to the toilet to promote independence urination, and in pharmaceutical support, such as by initiating or discontinuing treatment for dysuria. In this study, five cases in the adoption group required less frequent intermittent urethral catheterization per day. Although we did not follow the urinary clinical course of these cases, we considered that these results can be associated with improved quality of life in the patients.

Taken together, our results showed the positive effects of a pharmacist’s role in a urinary care team. However, we acknowledge certain limitations of this study. First, our results were based on retrospective and single-institutional data. Second, although, to our knowledge, this was the first study evaluating pharmaceutical support, the sample size was too small. Additional long-term prospective studies at multiple institutions are required to investigate a larger number of patients.

## Conclusion

Our findings suggested that pharmaceutical support by pharmacists in urinary care teams, in the form of recommendations for drug discontinuation or initiation, improved the outcome after catheter evulsion in patients with anticipated lower urinary tract disorders. We concluded that pharmacists are an important addition to urinary care teams.

## Abbreviations

ADL: activity of daily living
CI: confidence intervals
IQR: interquartile range
LUTS: lower urinary tract symptoms
ORs: odds ratios
